# Imbalanced Dopaminergic Transmission Mediated by Serotonergic Neurons in L-DOPA-Induced Dyskinesia

**DOI:** 10.1155/2012/323686

**Published:** 2011-10-11

**Authors:** Sylvia Navailles, Philippe De Deurwaerdère

**Affiliations:** Institut des Maladies Neurodégénératives, Université Victor Segalen Bordeaux 2, 33076 Bordeaux, France

## Abstract

L-DOPA-induced dyskinesias (LIDs) are one of the main motor side effects of L-DOPA therapy in Parkinson's disease. The review will consider the biochemical evidence indicating that the serotonergic neurons are involved in the dopaminergic effects of L-DOPA in the brain. The consequences are an ectopic and aberrant release of dopamine that follows the serotonergic innervation of the brain. After mid- to long-term treatment with L-DOPA, the pattern of L-DOPA-induced dopamine release is modified. In several brain regions, its effect is dramatically reduced while, in the striatum, its effect is quite preserved. LIDs could appear when the dopaminergic effects of L-DOPA fall in brain areas such as the cortex, enhancing the subcortical impact of dopamine and promoting aberrant motor responses. The consideration of the serotonergic system in the core mechanism of action of L-DOPA opens an important reserve of possible strategies to limit LIDs.

## 1. Introduction

Parkinson's disease is the second most devastating neurodegenerative disease affecting more than 6 million people worldwide and whose prevalence is expected to double within the next twenty years [1]. This neurological disorder is characterized by the progressive loss of mesencephalic dopaminergic (DA) neurons from the substantia nigra pars compacta and associated with numerous motor symptoms (bradykinesia, rigidity, and tremor) [2, 3]. L-DOPA, the precursor of DA, has been introduced in the mid 60's as a miracle pill to prevent the motor symptoms [4, 5]. However, upon chronic use of this medication, its efficacy slowly decreases leading to increase the doses of L-DOPA, which generate numerous side effects. After 5 to 10 years of L-DOPA treatment, Parkinsonian patients develop dyskinesias [6], which consist of stereotypical choreic or ballistic movements involving mostly the head, trunk, and limbs [7]. These abnormal involuntary movements are often more debilitating than the motor symptoms themselves. 

Preclinical research has permitted validating animal models to study the mechanisms of L-DOPA-induced dyskinesias (LIDs). The most commonly used rat model that shows best face and predictive validity, has been developed by Cenci and collaborators [8, 9] by producing severe lesion of the nigrostriatal DA pathway in adult rats with the unilateral injection of 6-hydroxydopamine (6-OHDA) in the medial forebrain bundle [10, 11]. A chronic treatment with L-DOPA for 3 weeks at low therapeutic doses (6–10 mg/kg) induced axial, limb, and orolingual abnormal involuntary movements (ALO AIMs) of variable occurrence and severity in rats [9, 12]. Despite extensive research done to understand how these motor complications develop in the Parkinsonian brain, all hypotheses could not be fully validated and new insights in this field need to be pushed forward to further gain in understanding of LIDs. In the present review, we will focus on the literature showing a prominent role of serotonergic neurons (5-HT) in the mechanisms of action of L-DOPA and how these neurons may contribute to the development of LIDs. Specifically, we will try to develop a new hypothesis that LIDs appear when the effect of L-DOPA falls in brain areas such as the cortex, then enhancing the subcortical impact of DA at the risk to elicit LIDs.

## 2. Mechanism of Action of L-DOPA in 5-HT Neurons and Collateral Consequences

It has long been thought that the therapeutic benefit of L-DOPA may depend on its ability to restore DA extracellular levels in the striatum through spared DA neurons [13–15]. However, contradictory data have shown that the fewer DA neurons that are spared, the more pronounced is the release of DA induced by L-DOPA [16–21]. Furthermore, L-DOPA-induced DA release is not sensitive to DA autoregulatory processes (DA-D2 autoreceptor stimulation and DAT blockade) [19]. Other monoaminergic cells [22, 23], namely serotonergic (5-HT) neurons, that are able to convert L-DOPA into DA, store and induce an exocytotic release of DA, rather participate in the mechanism of action of L-DOPA [24].

### 2.1. L-DOPA and 5-HT Neurons

5-HT neurons express the amino acid decarboxylase (AADC) that converts L-DOPA into DA and the vesicular membrane transporter VMAT2 that packages DA into exocytosis vesicles [25–28]. In line with these molecular features, 5-HT neurons have been shown for several years to release the newly synthesized DA from their cell bodies and terminals [25, 29, 30]. Indeed, 5-HT neurons are responsible for the TTX-sensitive, reserpine-sensitive, and DA drugs-insensitive release of DA induced by L-DOPA. The lesion of 5-HT neurons by the selective neurotoxin 5,7-DHT drastically reduces the increase in DA extracellular levels induced by a wide range of L-DOPA doses (3–100 mg/kg) [31, 32]. This effect is dependent on the extent of 5-HT denervation [31], which excludes the involvement of any other cellular system in the release of DA induced by L-DOPA. Furthermore, L-DOPA-induced DA release is sensitive to 5-HT autoregulatory mechanisms. Both the stimulation of 5-HT_1A_ autoreceptors by the 5-HT_1A_ agonist 8-OHDPAT [33] and the blockade of 5-HT transporters (SERT) by the selective serotonergic reuptake inhibitors (SSRI) fluoxetine [34] or citalopram [31] reduce the increase in L-DOPA-derived DA extracellular levels. These effects are thought to occur *via* the inhibition of 5-HT neuron activity [35–42]. Accordingly, it has been recently shown that high-frequency stimulation of the subthalamic nucleus, a surgical approach in Parkinson's disease able to inhibit 5-HT neuronal firing [43], also reduces L-DOPA-induced DA release [44].

**Figure 1:**
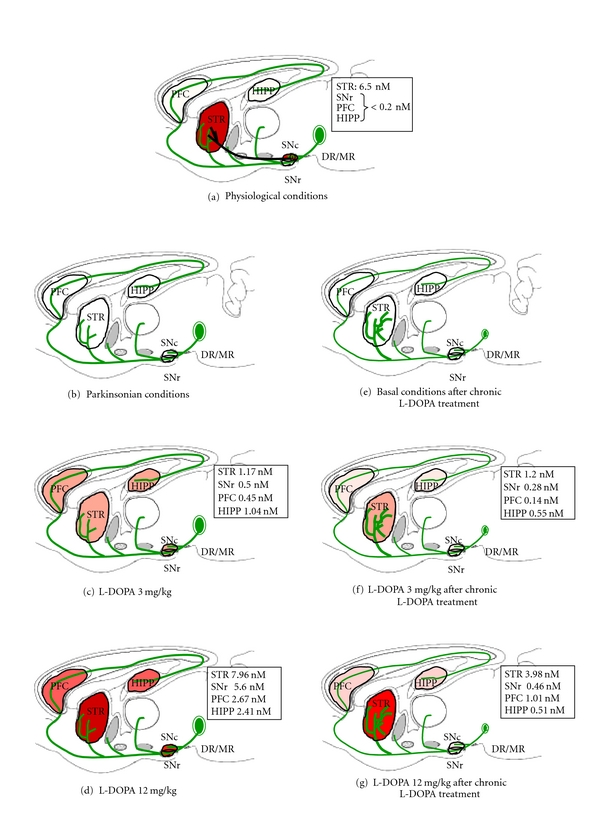
Serotonergic neurons are responsible for an imbalance of dopamine chemistry within brain regions in the Parkinsonian brain after acute and chronic L-DOPA treatment. Data taken from [31, 45].

 5-HT neurons send a widespread innervation from the raphe nuclei to the entire forebrain including the striatum [46, 47]. Beyond the increase in striatal DA extracellular levels, L-DOPA also induces a massive rise in DA levels in the prefrontal cortex (PFC), the substantia nigra pars reticulata (SNr), and the hippocampus (HIPP) [31]. In all brain regions, L-DOPA-induced DA release is sensitive to 5-HT pharmacological manipulation and the lesion of 5-HT neurons [31, 44, 48]. This ectopic release of DA induced by L-DOPA *via* 5-HT neurons creates a new balance in DA chemistry throughout the Parkinsonian brain (Figure 1) [24, 31]. In physiological conditions, basal DA concentrations are more than 30 times higher in the striatum compared to other brain regions, in line with the restricted innervation of mesencephalic DA neurons to striatal territories [5, 49]. While DA extracellular levels are from 4.6 to 7.8 fmol/uL, they are barely detectable depending on experimental conditions (below 0.2 fmol/uL) in the PFC, SNr, and HIPP although DA receptors are expressed [50]. In Parkinsonian conditions, the dose of L-DOPA required to “restore” similar DA concentrations in the DA-denervated striatum is about 12 mg/kg while it increases about 10 to 25 times DA concentrations in other brain regions (see Figure 1). Interestingly, L-DOPA at 3 mg/kg enhances DA levels to similar amounts (0.7 to 1.3 fmol) in the PFC, SNr, HIPP, and striatum. Therefore, huge amounts of DA can be released beyond the striatum [51] and may impact on DA receptors throughout the Parkinsonian brain. In keeping with the increased sensitivity of DA receptors that develops after DA denervation [52–54], such an imbalanced DA transmission between the striatum and other brain regions may participate in the emergence in both short-term benefits and long-term side effects of L-DOPA treatment (see Section 4). 

### 2.2. Chronic Impact of L-DOPA on DA Release Pattern in the Entire Forebrain

The therapeutic efficacy of L-DOPA treatment decreases over time with the development of numerous side effects including L-DOPA-induced dyskinesias (LIDs). LIDs are thought to emerge as a consequence of the dysregulated release of DA as a “false neurotransmitter” from 5-HT neurons [12, 31, 33, 44, 48, 57–63]. Indeed, the inhibition of L-DOPA-induced DA release by 5-HT_1_ autoreceptors stimulation [33, 48] and/or 5,7-DHT lesion [31, 32] is associated with a marked reduction in LIDs [48, 61]. However, these mechanisms have been described mostly in the striatum while other brain regions could be involved in the development of LIDs [60, 64–69]. Furthermore, the dose of L-DOPA used, even within the therapeutic range (3–12 mg/kg), represents a critical parameter to consider in the understanding of LIDs [70]. 

The occurrence and severity of LIDs in animals treated chronically with L-DOPA depend on numerous parameters, that is, the dose of L-DOPA, the site of 6-OHDA injection, the extent of DA lesion, and rat strain. About half of animals treated chronically with 3 mg/kg of L-DOPA develop LIDs. At 6 mg/kg, about 2/3 of the animals treated with L-DOPA display severe LIDs. At 12 mg/kg and above, almost all animals develop LIDs [9]. One consistent result observed after chronic L-DOPA treatment is that, whatever the dose, basal DA extracellular levels remain barely detectable in all brain regions. In our experimental conditions (12 mg/kg for 10 days), basal DA levels were below the detection limit in the striatum, SNr, PFC, and HIPP (Figure 1) [45]. In another study using a 14-day treatment with 6 mg/kg L-DOPA, baseline DA concentrations were reduced by 99% to 0.04 fmol/*μ*L in the striatum compared to intact animals (4 fmol/*μ*L) without changes in the SNr (0.1-0.2 fmol/*μ*L) [48]. One study showed a slight increase in basal DA levels in the striatum (reaching about 0.6 fmol/*μ*L) by using a higher dose (25 mg/kg) of L-DOPA administered twice a day [81]. 

Dynamics in the increase in DA release after each L-DOPA administration may, however, differ regarding the dose of L-DOPA used. Some authors have proposed that LIDs may emerge as a consequence of abnormal fluctuations in synaptic DA levels induced by L-DOPA treatment in dyskinetic animals [48, 58, 59, 61, 73]. Larger increases in synaptic DA levels induced by L-DOPA have been proposed to be responsible for the emergence of peak-dose dyskinesia in PD patients [59]. Data obtained with a chronic L-DOPA treatment at 6 mg/kg have shown that the kinetics of DA release are different in animals developing LIDs or not [73]. Although a higher magnitude of DA release was observed in the striatum and SNr of dyskinetic animals compared to nondyskinetic animals [48], this has not been consistently observed [73]. In a recent report, our data have provided new evidence for reconsidering the mechanisms of L-DOPA within the Parkinsonian brain and the putative consequences in many side effects including LIDs [45]. We showed that after a chronic L-DOPA treatment at 12 mg/kg for 10 days, the reactivity of 5-HT neurons to an acute challenge at 3 or 12 mg/kg of L-DOPA was modified and resulted in a potent loss of efficacy of L-DOPA to increase DA release (Figure 1). Most importantly, our data could depict a new imbalance created by chronic L-DOPA treatment within the striatum and other brain regions. The capacity of 5-HT neurons to increase DA release in the SNr, HIPP, and PFC was drastically reduced (about 70 to 90%) while it was less affected in the striatum. Indeed, the increase in striatal DA release induced by 3 mg/kg of L-DOPA after a 12 mg/kg treatment for 10 days was similar to that induced by an acute administration of 3 mg/kg. At 12 mg/kg, the effect of L-DOPA was reduced by only 50% after chronic compared to acute treatment. It appears that different mechanisms may be processed in the striatum compared to other brain structures that may account for the relatively preserved striatal DA effect of L-DOPA. Some of these mechanisms may be directly related to the specific features and heterogeneity of 5-HT terminals within brain regions. The resulted imbalance between cortical versus subcortical brain regions in DA transmission may potentially participate in development of LIDs.

The following paragraph corresponds to the description of the Figure 1, (a) in physiological conditions, dopaminergic neurons originating from the substantia nigra pars compacta (SNc) densely innervate the striatum (STR) where basal dopamine (DA) concentrations range between 4.6 and 7.8 nM. In the prefrontal cortex (PFC), the hippocampus (HIPP), and the substantia nigra pars reticulata (SNr), basal DA concentrations are much lower (<0.2 nM). All these brain regions express DA receptors and are innervated by serotonergic neurons that originate from the dorsal and medial raphe nuclei (DR/MR). (b) In Parkinsonian conditions (i.e., unilateral 6-hydroxydopamine lesion in rats, 6-OHDA rats), the neurodegeneration of DA neurons leads to undetectable levels of DA in any brain region examined. (c) In the absence of DA neurons, L-DOPA is decarboxylated into DA, stored into exocytosis vesicles, and released in the extracellular space by serotonergic neurons. In such physiopathological condition, an acute administration of L-DOPA at the low therapeutic dose of 3 mg/kg induces a homogeneous increase in DA concentrations in all brain regions (see values in the square box). These concentrations are 2, 2.5, and 5 times higher than in physiological conditions in the SNr, PFC, and HIPP, respectively, while they are 5 times lower in the STR. (d) An acute administration of L-DOPA at the moderate therapeutic dose of 12 mg/kg increases DA concentrations in the STR within the range of physiological values. Similar concentrations of DA are observed in the SNr and corresponded to >25 times the physiological concentrations. In the PFC and HIPP, DA concentrations are >10 times higher than in physiological conditions. (e) After a chronic L-DOPA treatment at a dose known to induce dyskinesias in all 6-OHDA rats (12 mg/kg/day for 10 days), basal DA concentrations remain below the detection limit in all brain regions. All biochemical 5-HT indexes (extracellular and tissue levels of 5-HT and its metabolite 5-HIAA) are decreased after chronic L-DOPA treatment, suggesting that 5-HT neurons suffer from chronic exposure to L-DOPA. Numerous data provide evidence for a 5-HT sprouting occurring specifically in the striatum [55, 56]. (f) After a chronic L-DOPA treatment (12 mg/kg/day for 10 days), a subsequent administration of 3 mg/kg L-DOPA is less efficient to increase DA release in the SNr, PFC, and HIPP compared to an acute administration of the same dose in L-DOPA-naïve 6-OHDA rats (see (c)). The ability of L-DOPA to increase DA levels is reduced by 43%, 68%, and 45% in the SNr, PFC, and HIPP, respectively. However, the efficacy of L-DOPA is not altered in the STR as DA levels reached similar concentrations in both L-DOPA-treated and L-DOPA-naïve 6-OHDA rats. (g) The ability of L-DOPA at 12 mg/kg to increase DA release is diminished in all brain regions after chronic L-DOPA treatment (12 mg/kg/day for 10 days). The highest loss of efficacy is observed in the SNr (−92%), then in the HIPP (−79%) and the PFC (62%). By comparison, the efficacy of L-DOPA remained mostly preserved in the STR (−50%), an effect that may be related to the striatal 5-HT hyperinnervation [55].

## 3. Changes in 5-HT Transmission Associated with L-DOPA Treatment

L-DOPA, by entering 5-HT neurons, mediates numerous changes in 5-HT neuron homeostasis [45]. The production of massive amounts of DA has tremendous impact on 5-HT function at the level of the metabolism, the activity, and the morphology of 5-HT neurons (Table 1). Changes in 5-HT indexes have been associated with the emergence of LIDs (Table 2). Such changes may represent critical indicators of the physiopathological state of the Parkinsonian brain that should be taken into consideration to better control 5-HT transmission and L-DOPA's side effects [24, 82, 83].

### 3.1. Impact of L-DOPA on 5-HT Transmission

Since the beginning of the 70's, numerous evidences started accumulating for an alteration of 5-HT neuron activity in response to L-DOPA (Table 1) [86–88]. The first report in 1970 by Ng et al. [25] showed that L-DOPA-enhanced [^3^H]-5-HT release from [^3^H]-5-HT preloaded midbrain slices. This increase in 5-HT release has been later confirmed *in vivo* by local administration of L-DOPA in the substantia nigra [71, 89]. The potent increase in 5-HT levels observed in these studies has been suggested to account for a nonexocytotic efflux of 5-HT *via* 5-HT transporters due to the strong displacement of 5-HT from exocytosis vesicles by the newly synthesized DA [24]. However, recent data using systemic administration of L-DOPA at moderate doses (3–12 mg/kg) have reported distinct effects on 5-HT release depending on the dose and the brain region dialysated. While an acute injection of 12 mg/kg L-DOPA decreases 5-HT extracellular levels in the PFC and SNr, a biphasic effect was observed in the HIPP and no effect in the striatum [44, 45]. A transient increase in 5-HT levels has been observed only after the very high dose of 100 mg/kg in all brain regions (Navailles et al., unpublished observation; see Table 1). Different mechanisms could be triggered regarding the dose of L-DOPA used (exocytotic versus nonexocytotic) while the region-dependent effects of L-DOPA on 5-HT release may reflect the anatomo-functional heterogeneity of 5-HT terminals [24]. 

After a chronic L-DOPA treatment (12 mg/kg/day for 10 days), the reactivity of 5-HT terminals to a subsequent challenge of L-DOPA (3–12 mg/gk) was further modified in a region-dependent manner [45]. The inhibitory effect of L-DOPA at 3 and 12 mg/kg on 5-HT release was potentiated in the SNr and HIPP of L-DOPA-treated rats but not in the PFC. In the striatum of L-DOPA-treated rats, 5-HT release remained unaltered by L-DOPA whatever the dose used. Interestingly, this region-dependent reactivity of 5-HT terminals appears to correlate with the ability of L-DOPA to increase DA release after a chronic treatment (see Section 2). Of particular interest, the lack of sensitivity of striatal 5-HT terminals to L-DOPA on 5-HT release is associated with a preserved increase in L-DOPA-induced DA release while the highest sensitivity of 5-HT terminals observed in the SNr leads to the most profound loss of efficacy of L-DOPA to increase DA release [45]. This imbalance between the striatum and the SNr could not be unmasked after a chronic treatment with L-DOPA at 6 mg/kg, which did not change the effect of L-DOPA on 5-HT release [48]. Nevertheless, it appears that, for a moderate though therapeutic dose of L-DOPA (12 mg/kg), its effects on DA transmission occur in detriment of 5-HT transmission [45]. The distinct molecular (variable expression and sensitivity to 5-HT_1A/1B_ receptors, SERT, VGLUT3, cation channels) [90–97], anatomical (originating from the medial or dorsal raphe nuclei) [46, 98], ontogenesis (pet1-dependent versus pet1-resistant 5-HT neurons) [99, 100] characteristics of 5-HT neurons projecting to these different brain regions may participate in this region-dependent changes in 5-HT and DA releases [24]. In addition, chronic L-DOPA treatment by itself also alters the morphology of these 5-HT neurons and the synaptic plasticity in various brain regions (Table 1) [55, 56, 101–104], an effect that may participate in the imbalanced 5-HT and DA transmissions within structures in the Parkinsonian brain and favor the onset of LIDs (see below). 

Beyond the changes of 5-HT release (Table 1), chronic L-DOPA also alters 5-HT transmission by modifying the expression and function of numerous 5-HT receptors. Studies that aimed at improving L-DOPA's effects have focused on 5-HT_1A/1B_ receptors. Although a decrease in 5-HT_1A_ receptor expression in the dorsal raphe (and hippocampus) [105, 106] may be directly linked to the loss of 5-HT neurons in Parkinsonian patients [107–111], an increase has been described in the neocortex of Parkinson's disease patients [112], the putamen [105], caudate nucleus, and middle layers of premotor-motor cortices of MPTP-treated monkeys [77]. Although it remains difficult to attribute these effects to the progression of the disease or to L-DOPA therapy in Parkinsonian patients [107, 113], a massive increase in 5-HT_1A_ receptor binding could be observed in the caudate nucleus of L-DOPA-treated MPTP-lesioned monkeys [77]. No alteration in 5-HT_1B_ binding has been observed in the striatum and substantia nigra of Parkinsonian patients [114] and 6-OHDA rats [115] while an increase in 5-HT_1B_ receptor expression in these brain regions has been reported after chronic L-DOPA treatment in 6-OHDA-lesioned rats [75]. Other 5-HT receptors such as 5-HT_2A_ and 5-HT_2C_ receptors have been proposed to improve L-DOPA therapy in Parkinson's disease [69, 79, 116–119]. These receptors are known to be sensitive to chronic alteration of DA transmission [119–122]. However, the few data available have reported conflicting results. 5-HT_2A_ receptor expression has been shown to increase in the striatum of 6-OHDA rats [76] and the neocortex of Parkinsonian patients [112] while it did not change in the putamen and PFC of MPTP-treated monkeys [78]. Although L-DOPA reversed the increase in the striatum of 6-OHDA rats [76], it increased 5-HT_2A_ receptor binding in the dorsomedial caudate nucleus of MPTP-treated monkeys [78]. 5-HT_2C_ receptor expression was decreased in the striatum but not in the subthalamic nucleus of 6-OHDA rats without any change after L-DOPA treatment [76]. However, the increased expression of 5-HT_2C_ receptors in the substantia nigra pars reticulata in Parkinsonian patients [80] appears to participate in the overactivity of this brain region by dampening the antiparkinsonian action of DA drugs in 6-OHDA rats primed with L-DOPA [120, 121]. Altogether, these data indicate that chronic L-DOPA treatment alters 5-HT function and the resulted changes in 5-HT markers have been mostly associated with the genesis and expression of LIDs.

### 3.2. Biochemical, Morphological, and Molecular Changes in 5-HT Indexes Associated with LIDs

Changes in 5-HT indexes have recently gained growing importance as they may reflect fluctuations in L-DOPA-induced DA release from 5-HT neurons that have been associated with the emergence of LIDs (Table 2) [61, 62]. In this attempt, most studies have focused on modifications of tissue and extracellular levels of 5-HT together with changes in 5-HT terminals density and morphology in 6-OHDA rats developing or not dyskinesias after a chronic treatment with 6 mg/kg of L-DOPA. Conflicting results, however, have emerged regarding 5-HT tissue and extracellular levels. Independently of the emergence of LIDs, chronic L-DOPA treatment either reduced [45, 48, 61], did not affect [72, 78], or tended to increase [84] 5-HT tissue and extracellular levels in the striatum. In most studies, however, tissue and extracellular 5-HT levels in the striatum and the cortex, but not the SNr, of rats developing LIDs were significantly higher than in nondyskinetic rats [48, 72, 84] suggesting a positive correlation to LIDs. Accordingly, Eskow et al. [85], by using selective 5,7-DHT lesions that are known to abolish both LIDs [61] and L-DOPA-induced DA release [31], could establish a positive correlation between striatal 5-HT levels and LIDs. These results are in contrast with the study by Gil et al. [72] in which 5-HT tissue levels were negatively correlated to axial, limb, and orolingual abnormal involuntary movements (AIMs). Interestingly, Carta et al. [84] could not establish a link between striatal 5-HT levels but did observe a positive correlation between 5-HT levels in the PFC and AIMs providing further evidence for a role of 5-HT function beyond the striatum in the emergence of LIDs.

In support of an increased 5-HT function in the genesis of LIDs, chronic L-DOPA treatment has been shown to increase AADC protein expression without increasing tyrosine hydroxylase expression in the lesioned-side striatum of dyskinetic rats [74]. This effect was associated with a higher 5-HT immunoreactivity compared to nondyskinetic animals, highlighting an increased 5-HT fiber density mediated by L-DOPA [74]. In line with this, Rylander et al. [55] have shown that chronic L-DOPA induced a dose-dependent increase in SERT-binding densities on the lesioned striatum (and motor-premotor cortices) that was associated with an increased number of striatal 5-HT varicosities but not with an increase in the number of 5-HT cell bodies or expression of SERT mRNA in raphe cells. Both striatal SERT binding and number of 5-HT varicosities correlated positively with the AIMs scores, showing that L-DOPA induced a sprouting of striatal 5-HT terminals in dyskinetic animals [55]. Furthermore, SERT-immunoreactive varicosities displayed larger synaptic incidence with striatal neurons and resulted in larger amount of stimulated (KCl evoked) [^3^H]-DA release in striatal slices from L-DOPA-treated dyskinetic rats [55]. However, Lundblad et al. [73] failed to correlate the higher 5-HT nerve density in the lesioned striatum of dyskinetic rats with the magnitude of KCl-evoked DA release measured *in vivo* by chronoamperometry after chronic L-DOPA treatment. Although SERT binding was decreased in the putamen and globus pallidus (GP) of MPTP-treated monkeys [55], a marked hyperinnervation of TPH-positive fibers (increase in number and diameter of TPH-positive axon varicosities) was observed in the dorsal caudate and putamen, but not the GP of MPTP-intoxicated monkeys [56]. Nevertheless, using both 5-HT markers, these studies have consistently shown an elevated SERT binding and a further increase in the number and enlargement of TPH positive axonal varicosities in caudate nucleus and putamen of MPTP-treated monkeys that develop LIDs [55, 56]. In Parkinsonian patients, SERT-binding levels were also significantly increased in both the putamen and GP of dyskinetic patients [55]. Regarding the lifetime L-DOPA medication, results indicate that patients with highest levels of SERT binding were those developing LIDs earliest during their PD treatment [55]. However, by using another marker of serotonin transporter (^11^C-DASP) in PET, Politis et al. [123] could not establish a correlation between ^11^C-DASP binding and exposure to dopaminergic therapy. Altogether, these data suggest that L-DOPA pharmacotherapy induced a maladaptive plasticity of 5-HT axon terminals that may predispose to LIDs. Indeed, the 5-HT hyperinnervation together with marked hypertrophy of 5-HT axon varicosities may worsen the fluctuations of L-DOPA-induced DA release [48, 55, 56, 59]. 

The combination of 5-HT_1A_ and 5-HT_1B_ agonists provides useful pharmacological manipulation to reduce the large increases in DA efflux and the occurrence of LIDs [48, 61, 124]. Their efficacy is reached when combining subthreshold doses of 5-HT_1A_ and 5-HT_1B_ agonists that are thought to activate presynaptic 5-HT_1_ receptors and dampen the release of L-DOPA-derived DA from 5-HT neurons [64]. The stimulation of postsynaptic 5-HT_1_ receptors on non-5-HT neurons may also contribute to their antidyskinetic effect by modulating GABA and glutamate release [64]. However, adverse effects involving the stimulation of postsynaptic 5-HT_1A_ receptors could worsen their therapeutic efficacy [125, 126]. Some studies have identified specific changes induced by chronic L-DOPA treatment on 5-HT_1B_ postsynaptic receptors that may be directly involved in the development of LIDs. Chronic L-DOPA treatment increased the expression of 5-HT_1B_ receptors and its adaptor protein p11 at striatonigral neurons [75]. The ability of 5-HT_1B_ agonist to reduce LIDs was p11 dependent [75]. Moreover, in L-DOPA-treated 6-OHDA rats that recovered from AIMs after a chronic treatment with citalopram (a selective serotonergic reuptake inhibitor, SSRI), the expression of 5-HT_1B_ receptors in the striatum was almost fully abolished [127]. The authors could reveal a positive correlation between the decreased anxiety induced by citalopram and its ability to reduce AIMs that involves a marked reduction in 5-HT_1B_ receptor expression (Table 2). However, in keeping with data obtained in the study by Zhang et al. [75], the reduction of LIDs by citalopram may not solely account for its effect on 5-HT_1B_ receptor expression but may also involve the ability of citalopram to abolish L-DOPA-induced DA release [31]. Nevertheless, these data allow identifying a new association between 5-HT_1B_ receptors and LIDs. A recent work could also highlight a relationship between 5-HT_2A_ receptors and LIDs in MPTP-treated monkeys [78]. [^3^H]Ketanserin-specific binding to 5-HT_2A_ receptors was increased in the dorsomedial caudate nucleus and anterior cingulated gyrus of dyskinetic L-DOPA-treated MPTP-intoxicated monkeys, an effect reversed by drugs inhibiting the expression of LIDs. The authors could reveal a positive correlation between the maximal dyskinesia scores at the end of L-DOPA treatment and 5-HT_2A_ receptor-specific binding in the anterior and posterior caudate nucleus as well as the nucleus accumbens [78].

Despite the high degree of variability observed in the changes of 5-HT markers across studies performed in different animal models and Parkinsonian patients, the available data to date allow establishing a clear role of the 5-HT system in the induction and maintenance of LIDs. The numerous 5-HT indexes used could provide interesting insights into the mechanisms of action of L-DOPA in mediating LIDs. However, the failure to fully correlate one change in 5-HT markers with a complex behavior such as LIDs may encourage future studies to reconsider the heterogeneity and the widespread influence of the 5-HT system as a whole fundamental index in the genesis of LIDs. Indeed, the numerous changes in 5-HT function induced by L-DOPA in multiple brain regions may concur in synergy to an imbalanced DA transmission that may participate in the emergence of LIDs.

## 4. Functional Outcomes of 5-HT Neuron-Mediated DA Transmission in LIDs

Because the 5-HT terminals are responsible for the gross increase in DA, leading to a homogeneous and ectopic release of DA in the brain, one may wonder the extent to which the striatum is involved in the therapeutic benefit of L-DOPA. It is far from our purpose to rule out many years of research centred on striatal DA transmission, but it is important to conceive that other brain regions play an important role in motor responses induced by L-DOPA. The main argument to look beyond the striatum is the success of the deep brain stimulation of the subthalamic nucleus in Parkinson patients, a surgical approach of the disease that does not rely on striatal DA release.

### 4.1. Role of Imbalanced Cortical-Subcortical DA Transmission in Motor Output

It is a common sense to reaffirm that DA transmission is altered in PD and that the relationships between DA transmission, symptoms severity, and medication coevolve with the deleterious progression of the disease. Nonetheless, adding the evidence that 5-HT neurons participate in the raise of extracellular DA offers a larger picture of the putative scenarios. In early stages of the disease, the presence of spared DA terminals and DAT in the striatum limits the excessive increase in DA extracellular levels induced by L-DOPA from 5-HT neurons. However, the increase in DA from 5-HT terminals in the striatum likely introduces a noise in the “coherent” DA transmission. Indeed, this aberrant release is not regulated while the “coherent” release from spared DA neurons is impaired due to the inhibitory effect of electrical activity of L-DOPA on DA neurons activity [19]. The more the disease progresses, the higher should be the contribution of 5-HT neurons in L-DOPA-induced DA release. Thus, even in the early stages of the disease, it is noticeable that L-DOPA is efficient to treat the core symptoms of the disease (tremor, bradykinesia, rigidity, posture) but has limited effects on precise coordinated movements or some impaired cognitive functions [128]. In the advanced stages of the disease, spared DA neurons are no longer able to buffer excessive swings of DA released from 5-HT neurons, a condition favoring motor fluctuations and LIDs [61].

Whatever the stage of the disease, the small release of DA from 5-HT neurons has potentially a larger impact beyond the striatum where DAT are poorly expressed. The impact may also be magnified due to altered pattern of activities found in extrastriatal territories such as the cortex or the HIPP. Indeed, numerous studies in humans using functional imaging have reported changes in activities in several cortical territories and the HIPP [129]. These brain areas expressing substantial amount of DA receptors [50], the excessive increase in DA release induced by L-DOPA in these territories could have a higher impact on the functions exerted by these brain regions. Of note, it has been described for many years that an increase in cortical DA may counteract aberrant DA signaling in subcortical areas. For instance, the catalepsy induced by the DA antagonist haloperidol, a rat model of Parkinsonism, is reversed by the direct infusion of DA into the PFC. Moreover, the increase in DA release induced by L-DOPA is very high in the SN, one of the brain regions receiving the strongest 5-HT innervation [46], and it has been shown for several years that the SN directly participates in the motor effects of L-DOPA in the 6-OHDA rat model of Parkinson's disease [68, 130].

The minimal release of striatal DA after therapeutic doses of L-DOPA could be compensated by an increase in D_2_ receptor efficiency. An increase in striatal D_2_ receptors has been reported in early stages of the disease, but some data have reported that DA “replacement” therapy reduced the excessive expression of striatal D_2_ receptors to levels comparable to matched controls [131, 132]. Based on the neurochemical data in the 6-OHDA rat model of Parkinson's disease, the benefit of L-DOPA could be an uneven release of DA or a hypodopaminergy in the striatum combined with an extrastriatal hyperdopaminergy.

### 4.2. Role of Imbalanced Cortical-Subcortical DA Transmission in LIDs

The increase in DA release induced by L-DOPA has been directly incriminated in LIDs [133]. Our data showing that chronic treatment with L-DOPA is associated with a dramatic loss of DA release in various rat brain areas compared to the striatum [45] points to an inverse schema. First, the inhibitory tone provided by cortical DA upon subcortical DA function would be lowered after chronic treatment, and subcortical DA release by 5-HT fibers would be preserved due to some sprouting of striatal 5-HT fibers [55, 56]. The situation is not known for several brain regions though it has been reported that LIDs in rodents is associated with an increase in c-Fos expression in the STN [134]. Besides, chronic L-DOPA treatment has been shown to increase c-Fos expression also in the cortex and globus pallidus [135, 136]. In addition, excessive DA tone in some brain regions other than the striatum may promote abnormal involuntary movement of the face, one clinical manifestation reported in LIDs in primates and rodents [7–9]. Second, the aberrant release of DA *via* 5-HT neurons would favor abnormal learning, at least in the striatum. Indeed, DA is critically involved in procedural learning, and LIDs is thought to result in part from aberrant molecular events at the level of medium spiny neurons of the striatum that involve DA receptors [104, 137, 138]. The postulated pathological form of synaptic plasticity may occur in the different territories of the striatum. It has been reported in MPTP-treated monkeys that LID involved not only the sensorimotor part of the striatum, but also its associative and limbic territories [139]. Third, as noted above, 5-HT processes could be involved as well [122, 140], particularly in considering that the “coherent” 5-HT transmission is altered by L-DOPA [24]. As for DA transmission, alteration in 5-HT transmission occurring elsewhere than the sensorimotor part of the striatum may promote abnormal movements in rodents [122, 141].

In a therapeutic point of view, one possibility is to limit the excessive DA transmission by administering a neuroleptic at risk of generating Parkinsonism. Nevertheless, the atypical neuroleptic clozapine has been shown to limit dyskinesia without aggravating the motor score [117]. It is difficult to interpret the origin of its efficacy as clozapine or other atypical antipsychotic drugs may slightly block subcortical DA transmission and enhance cortical DA transmission [142, 143]. Similar effects could account for the proposed efficacy of the antipsychotic and partial DA agonist drug aripiprazole [144]. According to the hypothesis above, treatment that is enhancing DA transmission in the cortex, that would limit the impact of cortico-subcortical glutamate transmission [145], could be a focus of future strategy against LIDs. It is noticeable that blockers of the N-methyl-d-aspartate receptor such as amantadine can also limit LIDs in patients [146]. 

The direct control of striatal DA transmission *via* 5-HT drugs is difficult to manage because the biochemical and behavioral relationships between 5-HT receptors and DA transmission are not well understood [147]. The use of 5-HT drugs able to control 5-HT nerve activity, to control the output of DA from 5-HT neurons, is a great opportunity, and clinical trials are currently underwent to alleviate LIDs using this strategy. The limit of this approach is that 5-HT drugs used may also act directly on cells other than 5-HT neurons due to the widespread distribution of 5-HT receptors in the brain. Also, a general decrease in DA release from 5-HT neurons may counteract dyskinesia and aggravate Parkinsonism [148–153]. Again, 5-HT drugs could be used to reinforce the initial imbalance created by L-DOPA, namely, the quite homogeneous pattern of DA release induced by L-DOPA, through cortical mechanisms.

## 5. Conclusion

The consideration of the 5-HT system in the core mechanism of action of L-DOPA opens many opportunities to better apprehend LIDs and to propose diverse therapeutic strategies in the treatment of LIDs. The excess of striatal DA released by L-DOPA remains an important preoccupation, but the possibility to facilitate DA transmission in the cortex could be also an interesting strategy. Additional studies are warranted to further study the imbalance of DA transmission promoted by the intervention of 5-HT neurons in the mechanism of action of L-DOPA to propose additional brain targets.

## Figures and Tables

**Table 1:** Changes in biochemical, morphological, and molecular 5-HT indexes in response to L-DOPA treatment.

Animal model	L-DOPA treatment	Biochemical 5-HT indexes	% of change	Reference
[^3^H]-5-HT preloaded rat	10 *μ*M	[^3^H]-5-HT release	+60%	[25, 29, 30]
midbrain slices				
naive rats	intra-SNr 5 *μ*M	*ext* 5-HT: STR and SNr	+55% in STR	[71]
			+102% in SNr	
6-OHDA rats	3, 6, 12, 100 mg/kg/d ip	*ext* 5-HT: STR, SNr, HIPP, PFC	**3:** *∅*	
			**6:** STR/PFC *∅*, SNr −22%,	[31] + unpublished observations
			HIPP −27%	
			**12:** STR/HIPP *∅*, SNr −17%,	
			PFC −27%	
			**100:** STR/SNr/HIPP *∅*, PFC −28%	
		*tiss* 5-HT: STR	**3–12:** *∅*	[31]
			**100:**−73%	
6-OHDA rats	12 mg/kg/d ip 14 d	*ext* 5-HT and 5-HIAA: STR, SNr, HIPP,	**5-HT:** STR −39%, SNr −45%,	[45]
		PFC	HIPP −29%, PFC –47%	
			**5-HIAA:** STR −32%, SNr −58%,	
			HIPP −44%, PFC −51%	
		*tiss* 5-HT and 5-HIAA: STR and CX	**5-HT:** STR −48%, CX −63%,	
			**5-HIAA:** STR −67%, CX −73%	
6-OHDA rats	6 mg/kg/d ip 14 d	*ext* 5-HT and 5-HIAA: STR and SNr	**5-HT:** STR-LID +125%,	
			STR-LND +75%; SNr-LID +104%, SNr-LND +108% **5-HIAA:** STR-LID −30%, STR-LND −73%; SNr-LID −28%, SNr-LND −37%	[48]
		*tiss* 5-HT and 5-HIAA: STR and SNr	**5-HT:** STR-LID −32%,
			STR-LND −78%
			5-**HIAA:** STR-LND −76%
6-OHDA rats	6 mg/kg/d ip 14 d	*tiss* 5-HT: STR	−48%	[61]
6-OHDA rats	6 mg/kg/d ip 21 d	*tiss* 5-HT: STR	+150%	[72]

Animal model	L-DOPA treatment	Morphological 5-HT indexes	% of change	Reference

6-OHDA rats	5 mg/kg/d ip 14 d	SERT immunoreactivity: STR	+266%	[73]
6-OHDA rats	6 mg/kg/d ip 21 d	5-HT immunoreactivity: STR	+70% in STR-LID	
			*∅* in STR-LND	[74]
6-OHDA rats	6 and 50 mg/kg/d ip 14–21 d	SERT-binding density: STR and CX	**6:** STR +37.5%, CX +75%	
			**50:** STR +87.5%, CX +125%	
		5-HT immunoreactivity: number of	**6:** +125%	
		varicosities, STR	**50:** +200%	[55]
		5-HT immunoreactivity: synapse	**6:** +155%	
		incidence in STR		
MPTP monkeys	Modopar (4 : 1) 15–20 mg/kg po	SERT-binding density: PUT and GP	PUT-LID +72%, GP-LID +400%, LND *∅*	
	6–8 m			[55]
MPTP monkeys	12.5 mg/kg/d po 1 m	TPH immunoreactivity: STR and GP	**STR:** increased number and size of varicosities and
			enlargement
			**GP:** enlargement in GPi/e + increased number of varicosities and length of fibres in GPe	[56]

Animal model	L-DOPA treatment	Molecular 5-HT indexes	% of change	Reference

6-OHDA mice and rats	(1) mice: 50 mg/kg/d ip 28 d	5-HT_1B_R binding: STR, GP and SNr	(1) STR +20%, GP *∅*, SNr +30%	
	(2) rat: 100 mg/kg 2×d ip 5 d (3) rat: 10 mg/kg/d ip 28 d		(2) STR +17%, GP +38%, SNr + 61% (3) STR +25%, GP *∅*, SNr +55%	[75]
6-OHDA rats	100 mg/kg 2×d ip 5 d	5-HT_1B_R protein: STR	+33%	[75]
6-OHDA rats	100 mg/kg 2×d ip 5 d	5-HT_2A_R mRNA: STR	−57%	
		5-HT_2C_R mRNA: STR and STN	*∅*	[76]
MPTP monkeys	Modopar	5-HT_1A_R-binding: STR, premotor-motor	acute: *∅*	
	acute: 14.6 mg/kg po	CX, HIPP	chronic: +140% in Caud matrix	[77]
	chronic: 14.6 mg/kg 2×d po 120 d			
MPTP monkeys	Prolopa 100/25 mg/kg po 1 m	5-HT_2A_R binding: STR and PFC	+58% in DM Caud	[78]
PD patients (LIDs)		5-HT_2C_R binding: SNr	+108%	[79, 80]

6-OHDA: 6-hydroxydopamine; MPTP: 1-methyl-4-phenyl-1,2,3,6-tetrahydropyridine; ip: intraperitoneal; sc: subcutaneous; po: oral; d: day; m: month; 2×d: twice a day; *tiss*: tissue; *ext*: extracellular; 5-HT: serotonin; 5-HIAA: 5-hydroxyindolacetic acid; AADC: amino acid decarboxylase; SERT: serotonergic transporter; 5-HT_1A_R: serotonin 1A receptor; 5-HT_1B_R: serotonin 1B receptor; 5-HT_2A_R: serotonin 2A receptor; 5-HT_2C_R: serotonin 2C receptor; STR: striatum; CX: cortex; PFC: prefrontal cortex; HIPP: hippocampus; SNr: substantia nigra pars reticulata; PUT: putamen; PFC: prefrontal cortex; STN: subthalamic nucleus; GPi/e: globus pallidus, internal/external part; DM Caud: dorsomedial caudate nucleus; LID: L-DOPA-treated dyskinetic animals; LND: L-DOPA-treated nondyskinetic animals; LIDs: L-DOPA-induced dyskinesias.

**Table 2:** Changes in biochemical, morphological, and molecular 5-HT indexes in dyskinetic (LID) *versus* nondyskinetic (LND) animals: correlation with dyskinesias (R).

Animal model	L-DOPA treatment	Biochemical 5-HT indexes	LID versus LND (R)	Reference
6-OHDA rats	6 mg/kg/d ip 14 d	*tiss* 5-HT: STR and CX	LID > LND R = 0.73 in CX	[84]
6-OHDA rats	12 mg/kg/d sc 5 d	*tiss* 5-HT (5,7-DHT): STR	R = 0.713	[85]
6-OHDA rats	6 mg/kg/d sc 14 d	*ext* and *tiss* 5-HT and 5-HIAA: STR and SNr	LID > LND in STR	[48]
6-OHDA rats	6 mg/kg/d ip 21 d	*tiss* 5-HT: STR	LID > LND R = −0.655	[72]

Animal model	L-DOPA treatment	Morphological 5-HT indexes	LID versus LND (R)	Reference

6-OHDA rats	5 mg/kg/d ip 14 d	SERT immunoreactivity: STR	LID > LND	[73]
6-OHDA rats	6 mg/kg/d ip 21 d	5-HT immunoreactivity and AADC levels: STR	LID > LND	[74]
6-OHDA rats	6 and 50 mg/kg/d ip 14–21 d	SERT-binding density: STR and CX	LID > LND, R = 0.796 in STR	[55]
MPTP monkeys	Modopar (4 : 1) 15–20 mg/kg po 6–8 m	SERT-binding density: PUT and GP	LID > LND in PUT	[55]

Animal model	L-DOPA treatment	Molecular 5-HT indexes	LID versus LND (R)	Reference

MPTP monkeys	Prolopa 100/25 mg/kg po 1 m	5-HT_2A_R-binding: STR and PFC	LID > LND in DM Caud and anterior cingulate gyrus	[78]

6-OHDA: 6-hydroxydopamine; MPTP: 1-methyl-4-phenyl-1,2,3,6-tetrahydropyridine; ip: intraperitoneal; sc: subcutaneous; po: oral; d: day; m: month; *tiss*: tissue; *ext*: extracellular; 5-HT: serotonin; 5-HIAA: 5-hydroxyindolacetic acid; 5,7-DHT: 5,7-dihydroxytryptamine; AADC: amino acid decarboxylase; SERT: serotonergic transporter; 5-HT_2A_R: serotonin 2A receptor; STR: striatum; CX: cortex; SNr: substantia nigra pars reticulata; PUT: putamen; PFC: prefrontal cortex; GP: globus pallidus; DM Caud: dorsomedial caudate nucleus; LID: L-DOPA-treated dyskinetic animals; LND: L-DOPA-treated nondyskinetic animals; R: correlation.
